# Effects of Upper Limb Control on the Less-Affected Side on Upper Limb Function, Respiration, Balance, and Activities of Daily Living in Stroke

**DOI:** 10.3390/medicina60060937

**Published:** 2024-06-03

**Authors:** Ju-O Kim, Mi-Young Lee, Byoung-Hee Lee

**Affiliations:** 1 Graduate School of Physical Therapy, Sahmyook University, Seoul 01795, Republic of Korea; k_juo@naver.com; 2 Department of Physical Therapy, Sahmyook University, Seoul 01795, Republic of Korea; mylee@syu.ac.kr

**Keywords:** less-affected side, stroke, upper limb control, upper limb function

## Abstract

*Background and Objectives:* This study aimed to investigate the effects of upper limb control exercises on upper limb function, respiration, balance, and activities of daily living in patients with stroke. *Materials and Methods:* The 28 patients who met the selection criteria were randomly assigned to two groups of 14 patients each. Subsequently, upper limb control exercises using real-time feedback were applied. The same interventional exercise was applied to both the less-affected and affected limbs of the study participants, who were classified into a less-affected side upper limb control group and an affected side upper limb control exercise group. Interventional exercises, 30 min each, were performed five times weekly for 4 weeks, and follow-up examinations were performed 2 weeks after the end of exercise. Electronic muscle strength measurements and an electronic goniometer were used to evaluate upper limb function. A spirometer was used to measure respiration. Balance ability was evaluated using a force plate pressure distribution measuring system with a sensor that detects the movement of the body center on the ground. Daily life movements were evaluated using the Korean version of the modified Barthel index. *Results:* When examining the results, the upper limb function on the paralyzed side showed an increase in the electromyographic strength of shoulder joint depression and flexion angle. Improvements were also observed in respiration (forced vital capacity [L] and forced expiratory volume in 1 s [L]), balance (95% confidence ellipse area [mm^2^] and center of pressure displacement [mm]), and daily life activities, all of which showed statistically significant differences in the time × group interaction effect (*p* < 0.05). *Conclusions:* Thus, it was found that the upper limb control exercise on the less-affected side had a significant effect when the exercise was performed together with treatment on the affected side in patients with stroke. It is anticipated that this study will provide basic data for evaluating both the trunk and upper limbs of the less-affected and affected sides.

## 1. Instruction

When a stroke occurs due to brain damage, severe motor impairments persist in the body, particularly on the opposite side of the brain, leading to muscle weakness and increased muscle tension. Involuntary or uncontrollable disorders, such as spasticity and associated reactions persist [1]. These aspects affect the non-paralyzed side of the brain and influence the ipsilateral neural pathways. Because of an imbalance in the body, the non-paralyzed side compensates for the inability of the paralyzed side to function, resulting in the overuse of the corresponding areas in the damaged hemisphere [2]. In particular, excessive tension in the trunk and upper limbs makes the precise movement of the upper limbs difficult, and it becomes challenging to achieve flexible movements of body segments during walking [3].

In patients with stroke, when upper limb movements occur on one side of the body while standing, precise and continuous posture adjustments are made to the contralateral trunk beforehand. Therefore, separate movements of the trunk and upper limbs are essential before movements of the arms and legs [1].

Furthermore, the feedforward system, which operates in conjunction with posture control during physical activity, is connected to the feedback system. This emphasizes the importance of information concerning the interactions between various systems within the body and the relationship between the body and its surrounding environment. In particular, when external information is provided, specific information conveyed by vision, proprioception, and the vestibular system is necessary for feedforward processes. This information enables posture control [4]. In patients with central nervous system damage, the trunk directs the body towards the central axis to maintain basic postural control. They adapt to tasks and environmental changes while standing against gravity. However, patients who experience hemiplegia due to stroke encounter difficulties in interaction and separate movements of the upper and lower limbs caused by impairments in trunk sensation and motor function [5]. In general, hemiplegia resulting from stroke typically manifests as muscle weakness and paralysis in the affected limbs [6]. However, observing damage location and functional changes in the trunk externally is not as straightforward [7]. Furthermore, trunk control ability is a crucial measure of functional recovery [8]. Therefore, to address visible limb synergies or weaknesses, separate parts of the trunk and upper limbs influence the overall aspects of the body [9].

Realignment of the body in patients with stroke can improve postural control and balance. However, in these patients, if the selective motor function of various trunk segments is impaired, the postural control function may be compromised. Therefore, for patients with stroke and hemiplegia, precise control of the trunk and limbs is necessary for postural control and normal functional activities [10].

Furthermore, patients with stroke commonly exhibit decreased balance abilities, such as asymmetry in the body, increased postural sway, and reduced weight-bearing capacity on the ground [5]. Balance ability is crucial for performing various functional body tasks, such as transitioning from sitting to standing positions [11]. Reduced balance ability resulting from impaired posture control in patients with stroke leads to an increased risk of falls, consequently causing secondary injuries such as fractures [12]. The majority of patients with stroke tend to support their weight predominantly on the non-paralyzed side while performing everyday activities such as standing or walking [13]. This phenomenon reduces the weight-bearing capacity of the affected limb during walking, leading to issues with body alignment and postural control. With repeated occurrences, this feedback loop can cause increased imbalance on the non-affected side due to overuse on the side of brain injury [6]. Excessive weight bearing on the non-paralyzed side can alter the feedforward pattern due to incorrect learning, leading to increased psychological fear in patients. This can restrict independent living and lead to dependency on caregivers [14]. Therefore, approaches aimed at improving postural control and balance ability after stroke are crucial for enhancing independent daily activities and addressing secondary issues [13].

Various approaches have been attempted to treat patients with stroke, including realignment of the trunk or upper limb function, changes in balance ability, task-oriented approaches, and alterations in breathing patterns [13,15,16]. Furthermore, patients with stroke can focus on movements of the paralyzed side and utilize interventions targeting the non-affected side to correct improper movements caused by hemiplegia [8].

Furthermore, through posture control training, it is possible to induce changes in areas where abnormal posture control due to brain injury has led to mismatches between the trunk and limbs, exacerbating asymmetry and impairing function [6]. Therefore, this study aimed to investigate the effects of non-paretic upper limb exercises on upper limb function, respiration, balance, and activities of daily living in patients with stroke.

## 2. Materials and Methods

### 2.1. Participants

The participants selected for this study were patients admitted to Hospital B in Suwon, South Korea.

Before recruiting participants for this study, we performed a power analysis using G*Power version 3.1.9.7 (Heinrich-Heine Universität, Düsseldorf, Germany). In the repeated-measures analysis of variance (ANOVA) and within–between interactions, an effect size f of 0.25 was obtained for all the outcome measures, with a α error probability of 0.05 to minimize type 1-β errors with a probability of 95%. The number of groups was two, and the number of measurements was five. As the estimated target sample size was 32, we recruited 45 participants who underwent physical therapy.

The study inclusion criteria were as follows: patients diagnosed with stroke with a duration of ≥6 months since onset; individuals with trunk and upper limb mismatch due to hemiplegia; patients without visual impairment; patients with a Korean Mini-Mental State Examination score of ≥24; patients capable of communication; patients whose evaluation was not limited by musculoskeletal disorders; and participants who understood and consented to this study. The exclusion criteria comprised patients who did not meet the inclusion criteria, those with communication difficulties or orthopedic surgical conditions, those with sensory impairment or severe pain, those with visual impairment or visual field defects, and those with dizziness.

All participants were provided with detailed explanations of the necessity and objectives of the study. Participants were informed that they could withdraw from the study at any time during the study period if they wished to do so. Subsequently, participants signed a consent form.

This study was approved by the Sahmyook University Institutional Review Board (approval number: 2-1040781-AB-N-01-2017030HR, approval date: 16 May 2017) and Clinical Research Information Service (KCT0005505). Participants fully understood the objectives and procedures used in the study. The study adhered to the ethical principles of the Declaration of Helsinki.

### 2.2. Experimental Procedure

The general characteristics of the 40 participants who met the inclusion criteria were assessed prior to the experiment. Sex, age, weight, and height were confirmed by questioning and measuring the participants and their guardians. To minimize errors related to the selection of groups and biases, the participants were randomly divided into two groups using the Research Randomizer program (http://www.randomizer.org/, accessed on 1 May 2017). They were then assigned to therapists to minimize bias.

All the participants were provided with a research schedule and participated in a training program. The participants were divided into an upper limb control of less-affected side (ULD-LA) group (14 participants) and an upper limb control of affected side (ULD-A) group (14 participants). The classification was based on the severity of motor impairments. Both groups performed trunk control exercises for 10 min, followed by the main exercise for 20 min. These sessions were conducted five times per week for 4 weeks. Both groups underwent follow-up assessments 2 weeks after treatment completion. This study involved six therapists responsible for interventions and four therapists responsible for evaluations. They conducted pretest, post-test, and follow-up assessments over three sessions.

The therapists underwent training on experimental safety and proper use of measurement equipment for 1 week before the commencement of pre-training and assessment. This was performed to minimize errors and ensure measurement accuracy. Throughout the study period, patients concurrently participated in general neurological rehabilitation therapy, typically provided during hospitalization, along with the intervention exercises outlined in this study.

#### Real-Time Feedback

Patients with stroke were permitted to adjust their height randomly using an elastic cord while supporting their pelvis on a wide sling in the supine position. The patients were instructed to raise their pelvis to a point marked by a physiotherapist on the wall using a laser point. Real-time feedback was provided verbally and visually by patients. This helped the patients maintain the posture of lifting the pelvis to a point marked on the wall.

### 2.3. Training Program

The upper limb control exercise training was conducted using a head-mounted display (HMD) (AR EYES, Smartpia, Seoul, Republic of Korea, 2012) to provide real-time feedback. This intervention exercise was implemented for a duration of 4 weeks. The exercise program was prerecorded and played back for the patients, with a video featuring real-time feedback displayed on both the HMD and laptop. The therapists applied the same program for both groups, divided into the less-affected side (ULD-LA) and the affected side (ULD-A), which consisted of posture control and upper limb exercise programs. For each week of the 4-week period, exercises for both the trunk and upper limbs were adapted and refined from the neurological rehabilitation exercises used by Kim et al. [17] for patients with stroke. These modified exercises were used in this study. The total duration of exercises spanning 4 weeks took place in a seated position, with each session lasting 30 min in the presence of a therapist (trunk control stabilization program: 10 min, ULD-LA program: 20 min). These sessions were conducted five times per week (Table 1, Table 2 and Table 3).

The aforementioned program is applied to the non-paralyzed side; when applied to the paralyzed side, it simply switches instructions for the paralyzed and non-paralyzed sides and applies them in the same manner.

### 2.4. Outcome Measurements

#### 2.4.1. Upper Limb Function Assessment

In this study, upper limb function was assessed using muscle strength, joint range of motion, and manual function tests. The muscle strength of the affected upper limb was measured using a Digital Manual Muscle Tester (Model 01163, West rexburg, USA, 2008). The evaluator straightened the participant’s arm and placed the assessment sensor on the opposite side of the flexion and extension to measure muscle strength. Measurements were performed thrice with a 1 min interval between each measurement to prevent fatigue, and the average value was used. The test–retest reliability of this assessment tool was r = 0.83, and the inter-rater reliability was ICC = 0.81 [18].

In this study, joint angles were measured using a digital goniometer (Digital Goniometer, Detroit, MI, USA, 2010). A digital goniometer was used to accurately measure the angles of shoulder joint flexion and extension with the acromial axis of the shoulder joint as the center. The range of motion of the affected side was measured while the patient was seated. Measurements were performed thrice with a 1 min interval between each measurement to prevent fatigue, and the average value was used. The test–retest reliability of this assessment tool was r = 0.81, and the inter-rater reliability was ICC = 0.79 [19].

Upper limb function was assessed using the manual function test, a tool for measuring motor ability and upper limb function in patients with stroke. It consists of eight items: movement (four items), grasping (two items), and finger manipulation (two items). The scores ranged from 0 (severe impairment) to 32 (complete functional ability). The test–retest reliability of this assessment tool was ICC = 0.86, and the inter-rater reliability was ICC = 0.91 [20].

#### 2.4.2. Respiratory Assessment

Respiratory function was assessed by using a spirometer (SPIROVIT SP-1, SCHILLER AG, Basel, Switzerland, 2002). Before the measurements, thorough explanations and demonstrations were provided to ensure that participants understood the procedure. Measurements were taken three times while the participant was in a seated position, with a 10 s rest period between each measurement, and the average value was used. The assessments were conducted in an upright position, and the following parameters were measured: forced vital capacity, forced expiratory volume in 1 s, and peak expiratory flow.

#### 2.4.3. Balance Assessment

In this study, balance assessment involved evaluating static balance ability in the standing position. A plantar pressure distribution measurement device (Zebris, Allgäu, Germany, 2015) was used to measure the difference in plantar pressure distribution. The pressure distribution measurement system from Zebris consisted of a mat approximately 60.5 cm high and 158 cm wide, equipped with 11,264 sensors. The pressure recorded at each pressure sensing point was transmitted through a conversion device and stored on a computer. The following parameters were evaluated and recorded: 95% confidence ellipse area of body sway, center of pressure (COP) path length, and COP average velocity. The test–retest reliability of this assessment tool was r = 0.81, and the inter-rater reliability was ICC = 0.91 [21].

#### 2.4.4. Activities of Daily Living

Activities of daily living were assessed using the Korean version of the modified Barthel index (K-MBI), a tool for evaluating basic activities of daily living through interviews with patients and caregivers. The K-MBI was adapted from the 5th edition of the MBI revised by Shah et al. [22] to better suit the Korean context. The total score of the K-MBI is out of 100 points, comprising a total of 10 items, each measured on a 5-point scale: 0–24 points indicate total dependence, 25–49 points indicate severe dependence, and 50–74 points indicate moderate dependence. Mild dependence is indicated by scores of 75–90 points, whereas minimal dependence is indicated by scores of 91–99 points, with 100 indicating complete independence. The test–retest reliability of this assessment tool is r = 0.88, and the inter-rater reliability is ICC = 0.95 [23].

### 2.5. Data Analysis

All statistical analyses were performed using SPSS version 20. Descriptive statistics, including means and standard deviations, were calculated for the general characteristics of the participants and experimental data. Normality testing was performed using independent t tests and statistical analyses were performed accordingly. Additionally, the patients selected as research participants were divided into two groups: the less-affected (non-paretic side) and affected (paretic side) upper limb exercise group. This study investigated upper limb function, respiration, balance, and activities of daily living using repeated-measures ANOVA to examine the effects of time, group, and the interaction between group and time. The statistical significance level for all data was set at 0.05.

## 3. Results

### 3.1. General Characteristics of the Participants

Among the participants, the general characteristics of the upper limb control of less-affected side Group and the upper limb control of affected side Group were found to be homogeneous (Table 4).

### 3.2. Changes in Upper Limb Function of Affected Side

In the within-group analysis, when examining the changes in the upper limb function of the affected side according to measurement time, statistically significant differences were observed in the muscle strength of shoulder extension and the joint range of motion of shoulder extension over time (*p* < 0.05). Shoulder flexion muscle strength, shoulder extension muscle strength, and shoulder flexion range of motion in the manual function test were significantly different between the groups (*p* < 0.05). Table 5 indicates a statistically significant difference in the interaction effect between time and group for shoulder extension muscle strength and range of motion of shoulder flexion (*p* < 0.05) (Table 5).

### 3.3. Changes in Respiration Function

In the within-group analysis examining the changes in respiration according to measurement time, forced vital capacity (L) and forced expiratory volume in one second (L) showed a statistically significant difference in the interaction effect between time and group (*p* < 0.05). A significant difference in peak expiratory flow was observed between the groups (Table 6).

### 3.4. Changes in Balance

In the within-group analysis examining the changes in balance ability according to measurement time, the 95% confidence ellipse area (mm^2^) and COP path length (mm) showed statistically significant differences in the effects of time, group, and the interaction between time and group (*p* < 0.05). However, the average COP velocity (mm/s) showed a significant difference only with time (*p* < 0.05) (Table 7).

### 3.5. Changes in Activities of Daily Living

In within-group effect tests examining changes in everyday actions depending on the measurement period, statistically significant differences were observed in the time (time), group (group), and time × group interaction effects (time × group interaction) (*p* < 0.05) (Table 8).

## 4. Discussion

Numerous studies have been conducted on both the paralyzed and nonparalyzed upper limbs in patients with stroke to investigate changes in posture and increased muscle efficiency [6,8,24]. Chan et al. [18] investigated the effects of exercising the upper limbs of patients with stroke on the function of the paralyzed upper limbs. The active range of motion of shoulder flexion and upper limb function were measured. In patients with moderate severity, a significant improvement was observed (*p* < 0.05), whereas no significant difference was observed in patients with mild severity. Providing assistive devices to the upper limbs of patients with stroke was effective in improving functional movements such as shoulder flexion. However, the effects on upper limb function in patients with mild disease were not discernible.

In this study, we found a time × group interaction effect on shoulder abduction isokinetic strength (lb) and shoulder flexion angle (degrees). Arya et al. [24] investigated the effects of limb-based mirror therapy on motor and functional recovery in chronic post-stroke hemiparetic patients. This study involved 36 participants who underwent functional movements using both the upper and lower limbs to induce and improve the function of the paralyzed side using the non-paralyzed side. Exercises involving the non-paralyzed side led to improved upper limb function, which aligns with the findings of studies conducted by Arya et al. [24] and Silva et al. [25], in which upper limb exercises induced postural control. Exercises involving the non-paralyzed side induce efficient movement of the trunk and upper limbs, leading to increased upper limb strength and shoulder joint angles. Baker et al. [26] demonstrated that core stabilization exercises resulted in changes in paralyzed upper limbs, with a significant between-group effect observed for shoulder flexion (*p* < 0.05). Furthermore, the core stabilization exercise program was helpful in reducing the overuse of trunk and shoulder movements in patients with stroke. This is consistent with the observations made in the study by Silva et al. [25], in which an increase in the strength of the non-paralyzed upper limb through training led to an improvement in upper limb function, which was observed through separate movements of the trunk and upper limbs.

Improved postural control ability through intervention exercises may help regulate the non-paralyzed side, thereby enhancing the function of the paralyzed side. In this study, when examining changes in respiration in patients with stroke, forced vital capacity (L) and forced expiratory volume in one second (L) showed statistically significant differences in the time × group interaction effect (*p* < 0.05). This improvement may be attributed to the improvement in stroke-induced trunk muscle inactivation through core stabilization and upper limb control exercises, as observed in a study by Schneemilch et al. [27], leading to respiratory improvement through chest expansion. Jandt et al. [28] investigated the correlation between the respiratory muscles in patients with stroke. They evaluated and measured the correlations among lung function, respiratory muscle strength, and trunk control in 23 participants. A statistically significant correlation was observed between trunk control ability and peak expiratory flow rate and between trunk control ability and peak expiratory pressure. A correlation was observed between trunk control ability and respiratory muscle strength (*p* < 0.05). The efficient postural control of the trunk and upper limbs observed in the studies by Lipska et al. [29] and Jandt et al. [28] is believed to have resulted in increased lung capacity through chest expansion. Lipska et al. [29] investigated the effects of a 16-week trunk stabilization breathing exercise program on patients with stroke. Although no significant differences were observed in lung function, respiratory muscle strength, general strength, aerobic capacity, quality of life, and lung capacity after 4 weeks of evaluation, there was an increase of between 20.54% and 27.6% in maximum expiratory pressure and maximum inspiratory pressure. Respiratory exercises had a positive impact on muscle strength, quality of life, and overall health. This refers to the studies conducted by Lipska et al. [29] and Themudo et al. [30], who implemented trunk or upper limb control exercises in patients with stroke. In this study, we concluded that implementing non-paretic upper limb control exercises in the experimental group resulted in enhanced respiratory capacity by reducing excessive tension in the non-paretic trunk and upper limbs, consequently facilitating thoracic expansion in patients with stroke.

When examining the balance changes according to postural changes using trunk and upper limb exercises in patients with stroke, Krukowska et al. [21] found significant alterations in COP (center of pressure) displacement among the four groups through a comprehensive approach to neurological rehabilitation therapy (*p* < 0.05). Significant differences were observed in the COP displacement distance and velocity among the four groups (*p* < 0.05). Park et al. [31] conducted a study on patients with stroke involving feedback training based on foot position, in which the COP displacement decreased from 241 to 188 mm, whereas the COP mean velocity decreased from 25 to 13 mm/s. Therefore, there was a significant improvement in balance ability before and after the intervention in the experimental group compared to the control group (*p* < 0.05). Kanekar et al. [32] found a significant improvement (*p* < 0.05) in lower limb muscle activation and efficient posture maintenance due to trunk posture control exercise programs, indicating enhanced efficiency in posture control. In this study, statistically significant differences were observed in the 95% confidence ellipse area (mm^2^) and the distance of the center of mass displacement (mm) in terms of time, group, and time × group interaction effects (time × group interaction) (*p* < 0.05). Similar to the findings of Kanekar et al. [32], stabilizing the trunk and conducting upper limb control exercises improved lower limb muscle functionality in crucial positions, thereby enhancing overall body control. Furthermore, it can be inferred that improving anticipatory postural control through the realignment of the trunk and upper limbs, as demonstrated in the studies by Krukowska et al. [21] and Pandian et al. [8], contributes to enhanced balance ability in the standing posture of patients with stroke.

In the within-group effect analysis, statistically significant differences were observed in time, group, and the interaction effect of time and group (time × group interaction) regarding changes in daily movements based on the measurement period (*p* < 0.05). Similar to the study by Kang et al. [33], providing visual feedback through upper limb control exercises helps regulate overused upper limbs, stabilize the trunk, and increase body balance ability, consequently enhancing performance in everyday activities.

This study included a limited number of patients with stroke and employed exercise methods focusing on trunk and upper limb interventions for both the less-paretic and paretic sides. Also, the time of intervention and the effect were limited to chronic patients with stroke mainly in the cerebral middle artery. Therefore, supplementary research is necessary to explore complementary exercises targeting different body areas in addition to these interventions.

Generally, when treating patients with stroke in clinical settings, efforts are made to address issues such as hypotonia, hypertonia, and muscle weakness in the affected areas of paralysis. A significant role is also attributed to areas that regulate the upper brainstem, such as the corticospinal tract, which symmetrically descends from the brain beneath the cortex. However, without addressing the involvement of the descending pathways from the brainstem to the trunk and upper and lower extremities, such as the vestibulospinal and reticulospinal tracts descending ipsilaterally, stability and postural control in the trunk and extremities cannot be adequately managed. Failure to address these pathways can impede the resolution of fundamental issues. Therefore, this study focused on non-paretic areas that are closer to the actual site of brain damage. During the 4-week experimental period, the non-paretic upper limb control exercise group did not effectively regulate the overuse of the non-paretic side during daily activities outside of the treatment sessions in the hospital room.

## 5. Conclusions

This study examined the effects of nonparetic upper limb control exercises in patients with stroke. Twenty-eight participants were divided into two groups: non-paretic and paretic upper limb control exercise groups, each consisting of 14 patients. The intervention exercises were conducted five times per week for 30 min over 4 weeks, with follow-up assessments performed 2 weeks after the end of the study. An analysis of the results revealed significant changes in the function of the paretic upper limb, including improvements in scapular elevation muscle strength, shoulder flexion joint angle, respiration (forced vital capacity [L], forced expiratory volume in 1 s [L]), balance (95% confidence ellipse area [mm^2^], distance of the center of mass displacement [mm]), and the interaction effect of time and group on daily activities, showing statistically significant differences (time × group interaction) (*p* < 0.05). Hence, in clinical practice, when treating hemiplegic patients with stroke, it is anticipated that addressing the nonparetic side, which is related to brain injury on the ipsilateral side, alongside conventional treatments targeting the affected side, will improve movement efficiency and result in more effective actions.

## Figures and Tables

**Table 1:** Trunk control stabilization program.

Sequence	Intermediate and Finishing Postures
1	Seat the patient on the mat and stabilize their feet and ankles near the midline.
2	Hold the pelvis and grasp the proximal femurs to stabilize them near the midline. Perform steps 1 and 2 bilaterally.
3	Place both hands on the table and align the trunk with the midline.
4	The therapist stabilizes the pelvis by holding it with both hands.
5	The therapist holds the patient’s lower trunk and performs trunk extension.
6	The therapist holds the patient’s lower trunk and performs trunk flexion.
7	The therapist holds the patient’s trunk and moves toward the affected side.
8	Therapist holds the patient’s trunk and moves toward the non-affected side before stabilizing in the center.

**Table 2:** Upper limb control exercise training using a head mounted display (HMD).

1	The patient wears the HMD and prepares on the mat.
2	Prepare for trunk control exercise program (10 min).
3	The therapist holds the patient’s trunk and upper limb on the affected side and waits.
4	Play the intervention video with real-time feedback from the HMD connected to the laptop, along with the verbal command from the assistant to “start”.
5	The patient follows the movements while watching the video on the HMD, and the therapist conducts treatment while adjusting the posture on the affected side (20 min).
6	Treatment focuses on enhancing posture control on the affected side and promoting mobility of the upper limb. This is achieved by adjusting the posture of the trunk and upper limb on the affected side, leading to improved stability on the non-affected side and increased mobility of the upper limb.

**Table 3:** Upper limb control exercise program.

Weeks	Intermediate and Finishing Postures	Picture
1st week	A. Stabilize the trunk and secure the paralyzed arm on the assistive table. B. Flex and extend the fingers of the non-paralyzed side to regulate tension. C. Repeat the movement of flexing and then extending the wrist of the non-paralyzed side. D. Repeat the movement of pronating and then supinating the wrist of the non-paralyzed side. E. All movements are performed in collaboration with the therapist, who assists in controlling the patient’s upper limb strength while actively participating.	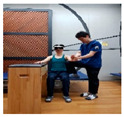
2nd week	A. Stabilize the trunk and secure the paralyzed arm on the assistive table. B. Flex the elbow of the non-paralyzed side and then extend it again. C. Bend the elbow of the non-paralyzed side and then flex the shoulder joint. D. Bend the elbow of the non-paralyzed side and then bring the shoulder joint forward. E. All movements are conducted with a therapist, actively assisting while regulating the patient’s upper limb strength.	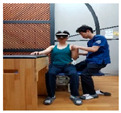
3rd week	A. Stabilize the trunk and secure the paralyzed arm on the assistive table. B. In a flexed position of the non-paralyzed elbow, move the shoulder joint backward. C. In a bent position of the paralyzed elbow, move the shoulder joint upward. D. In a flexed position of the non-paralyzed elbow, move the shoulder joint downward. E. All movements are conducted with a therapist, actively assisting while regulating the patient’s upper limb strength.	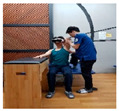
4th week	A. Stabilize the trunk and secure the paralyzed arm on the assistive table. B. In the bent position of the non-paralyzed elbow, flex the shoulder joint upward. C. In the bent position of the non-paralyzed elbow, flex and extend the upper limb. D. In the bent position of the non-paralyzed elbow, flex and extend the lower limb. E. All movements are conducted with a therapist, actively assisting while regulating the patient’s upper limb strength.	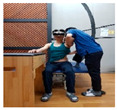

**Table 4:** General characteristics of participants (*n =* 28).

Characteristics	ULD-LA Group (*n* = 14)	ULD-A Group (*n* = 14)	*t* (*p*)
Sex (M/F)	9/5	6/8	−1.520 (0.617)
Age (years)	61.15 (1.61)	63.32 (4.21)	1.886 (0.637)
Height (cm)	167.64 (7.92)	161.42 (5.15)	2.213 (0.334)
Weight (kg)	67.86 (12.60)	66.54 (2.93)	1.746 (0.945)
Lesion sites (Rt/Lt)	7/7	7/7	0.366 (1.000)
Onset period (month)	10.71 (4.23)	12.57 (5.15)	1.325 (0.617)

*n* (%); M (SD); Rt: right side; Lt: left side; ULD-LA: upper limb control of less-affected side; ULD-A: upper limb control of affected side.

**Table 5:** Changes in upper limb function of the affected side (*n =* 28).

		ULD-LA (*n* = 14)	ULD-A (*n* = 14)	Time F (*p*)	Group F (*p*)	Time × Group F (*p*)
A	0 w	70.12 (18.15) ^a^	60.61 (11.25)	3.072 (0.091)	11.426 (0.002) *	0.296 (0.591)
	4 w	74.62 (20.14)	61.62 (13.67)			
	6 w	78.93 (21.66)	62.63 (10.85)			
B	0 w	61.92 (17.18)	53.48 (20.13)	10.234 (0.004) *	9.954 (0.004) *	0.113 (0.039) *
	4 w	62.56 (18.53)	55.04 (18.44)			
	6 w	69.28 (17.94)	54.47 (16.59)			
C	0 w	112.32 (43.58)	87.43 (51.53)	2.782 (0.107)	5.397 (0.028) *	4.635 (0.041) *
	4 w	127.42 (45.40)	88.13 (52.01)			
	6 w	130.32 (41.94)	89.13 (48.55)			
D	0 w	36.14 (17.33)	32.91 (15.68)	5.455 (0.027) *	3.388 (0.077)	2.343 (0.138)
	4 w	41.64 (15.22)	31.21 (31.22))			
	6 w	50.54 (34.81)	38.01 (19.83)			
E	0 w	17.62 (9.63)	17.91 (10.22)	1.064 (0.312)	0.894 (0.033) *	1.064 (0.312)
	4 w	21.21 (8.68)	18.41 (9.77)			
	6 w	22.42 (8.88)	17.94 (9.15)			

^a^ M (SD). ULD-LA: upper limb control of less-affected side; ULD-A: upper limb control of affected side; A: muscle strength of shoulder flexion (lb); B: muscle strength of shoulder extension (lb); C: joint range of motion of shoulder flexion (degrees); D: joint range of motion of shoulder extension (degrees) E: manual function test (score); 0 w: before training (0 weeks); 4 w: after training (4 weeks); 6 w: follow-up assessment (6 weeks); * *p* < 0.05.

**Table 6:** Changes in respiration function (*n =* 28).

		ULD-LA (*n =* 14)	ULD-A (*n =* 14)	Time F (*p*)	Group F (*p*)	Time × Group F (*p*)
A	0 w	3.14 (0.43) ^a^	2.68 (1.78)	1.180 (0.287)	0820 (0.374)	6.007 (0.021) *
	4 w	3.34 (0.36)	2.07 (1.65)			
	6 w	3.94 (0.49)	2.56 (0.24)			
B	0 w	1.83 (0.19)	1.28 (0.24)	1.465 (0.237)	3.296 (0.081)	8.696 (0.007) *
	4 w	1.84 (0.16)	1.27 (0.13)			
	6 w	2.13 (0.21)	1.36 (0.44)			
C	0 w	2.61 (0.39)	2.18 (0.40)	2.225 (0.148)	6.813 (0.015)	2.245 (0.146)
	4 w	3.11 (0.45)	2.17 (0.16)			
	6 w	3.41 (0.45)	2.46 (0.27)			

^a^ M (SD). ULD-LA: upper limb control of less-affected side; ULD-A: upper limb control of affected side; A: forced vital capacity (L); B: forced expiratory volume in one second (L); C: peak expiratory flow (L/min.); 0 w: before training (0 weeks); 4 w: after training (4 weeks); 6 w: follow-up assessment (6 weeks); * *p* < 0.05.

**Table 7:** Changes in balance (*n =* 28).

		ULD-LA (*n* = 14)	ULD-A (*n* = 14)	Time F (*p*)	Group F (*p*)	Time × Group F (*p*)
A	0 w	1057.86 (149.11) ^a^	1096.11 (171.52)	12.776 (0.001) *	5.275 (0.030) *	12.741 (0.035) *
	4 w	1010.54 (186.41)	1183.28 (210.54)			
	6 w	919.22 (155.63)	1066.21 (191.23)			
B	0 w	916.12 (157.71)	1271.22 (101.64)	30.504 (0.000) *	7.753 (0.010) *	0.273 (0.042) *
	4 w	793.01 (161.41)	1216.23 (119.14)			
	6 w	734.09 (161.63)	1211 (121.16)			
C	0 w	19.21 (3.56)	27.94 (2.05)	0.767 (0.000) *	1.168 (0.290)	1.787 (0.193)
	4 w	17.99 (27.48)	64.67 (19.52)			
	6 w	16.64 (23.04)	58.21 (16.43)			

^a^ M (SD). ULD-LA: upper limb control of less-affected side; ULD-A: upper limb control of affected side; A: 95% confidence ellipse area (mm^2^); B: COP path length (mm); C: COP average velocity (mm/s); 0 w: before training (0 weeks); 4 w: after training (4 weeks); 6 w: follow-up assessment (6 weeks); * *p* < 0.05.

**Table 8:** Changes in activities of daily living (*n =* 28).

		ULD-LA (*n* = 14)	ULD-A (*n* = 14)	Time F (*p*)	Group F (*p*)	Time × Group F (*p*)
KMBI	0 w	74.31 (3.58) ^a^	65.06 (2.64)	2.747 (0.044) *	3.003 (0.035) *	0.277 (0.03) *
	4 w	77.41 (4.51)	70.66 (2.27)			
	6 w	79.19 (3.05)	70.97 (3.19)			

^a^ M (SD). ULD-LA: upper limb control of less-affected side; ULD-A: upper limb control of affected side; KMBI: Korean version of the modified Barthel index; 0 w: before training (0 weeks); 4 w: after training (4 weeks); 6 w: follow-up assessment (6 weeks); * *p* < 0.05.

## Data Availability

Data are contained within the article.
